# The changes in brain network functional gradients and dynamic functional connectivity in SeLECTS patients revealing disruptive and compensatory mechanisms in brain networks

**DOI:** 10.3389/fpsyt.2025.1584071

**Published:** 2025-05-09

**Authors:** Linfeng Song, Guangrong Wu, Jiaren Zhang, Benqing Liu, Xu Chen, Junjun Wang, Xiaoyu Gu, Binlin Tian, Yongzhe Li, Anjie Zhang, Xuejin Ma, Lin Jiang

**Affiliations:** Department of Radiology, The Third Affiliated Hospital of Zunyi Medical University (The First People’s Hospital of Zunyi), Zunyi, China

**Keywords:** self-limited epilepsy with centrotemporal spikes, functional gradient, dynamic functional connectivity, brain network, machine learning

## Abstract

**Background:**

Self-limited epilepsy with centrotemporal spikes (SeLECTS), a common childhood focal epilepsy syndrome, is linked to cognitive impairments and poorly understood neuropathological mechanisms.

**Methods:**

This study explored dynamic functional connectivity (dFC) and functional gradients (FG) alterations in key brain networks using resting-state MRI (rs-MRI) data from 34 SeLECTS patients and 32 healthy controls (HCs).

**Results:**

The results revealed significant dFC changes between the Default Mode Network (DMN) and Sensorimotor Network (SMN) in SeLECTS patients compared to HCs. Specifically, the first gradient of the DMN showed decreased gradient scores in the bilateral dorsolateral superior frontal gyrus and increased scores in the left inferior temporal gyrus. In the first gradient of the SMN, increased scores were found in the bilateral supplementary motor area, while decreases occurred in the right precentral gyrus. Support vector machine (SVM) analysis showed that FG-based features could effectively identify abnormalities in specific brain networks of SeLECTS (AUC = 0.819). Further correlation analysis linked FG alterations in the DMN to cognitive measures (working memory, processing speed, and full-scale IQ) and in the SMN to disease duration and language comprehension.

**Conclusion:**

These findings suggest that significant changes in FG and dFC of DMN- and SMN-related brain regions in SeLECTS may reflect both disruptions and compensatory mechanisms in brain networks, offering new insights into the neuropathological basis of the disorder and potential diagnostic biomarkers.

## 1 Introduction

Self-limited epilepsy with centrotemporal spikes (SeLECTS) is one of the most common focal epilepsy syndromes in children, accounting for approximately 6-7% of pediatric epilepsy cases (1, 2). Although the seizure symptoms typically diminish in adolescence, an increasing body of evidence suggests that children with SeLECTS exhibit deficits in higher cognitive functions such as attention, executive function, and memory (3, 4). These impairments can significantly affect academic performance and daily activities and are often related to the disruption of critical brain networks by epileptic discharges (5, 6). However, the neurobiological mechanisms underlying these cognitive deficits are not fully understood, especially in terms of how brain network and functional connectivity abnormalities contribute to cognitive dysfunction. Therefore, investigating the brain network changes, functional connectivity abnormalities, and their relationship to cognitive functions in SeLECTS provides a new perspective for understanding its neurobiological mechanisms.

Resting-state functional MRI (rs-fMRI) has been widely applied in the study of neurological and psychiatric disorders, including Alzheimer’s disease, psychosis, and epilepsy, revealing abnormal brain network activity in these conditions (7–9). Research indicates that children with SeLECTS exhibit disruptions in both static functional connectivity (sFC) and dynamic functional connectivity (dFC), affecting multiple key brain networks. For instance, epileptic discharges in SeLECTS predominantly originate in the Rolandic area, leading to abnormalities in the sensorimotor network (SMN), which may impair motor control and sensory processing (10, 11). Furthermore, dysfunctions in the frontoparietal control network (FPN) and dorsal attention network (DAN) suggest deficits in executive function and attention regulation (6). Task-based fMRI studies have provided additional insights into altered brain network function in SeLECTS during cognitive tasks. Notably, reduced default mode network (DMN) activation during working memory and language tasks suggests impaired functional modulation of task-relevant regions (12). The DMN is typically active at rest but is suppressed during cognitive engagement. In SeLECTS, impaired suppression of the DMN may lead to weakened modulation by the FPN, consequently affecting executive function, language processing, and attention control (13). Additionally, some studies have identified increased functional connectivity between the DMN and SMN in children with SeLECTS, which may reflect a compensatory mechanism aimed at preserving essential cognitive functions (14). Despite these advances, several research gaps remain. First, most studies have emphasized sFC, while relatively few have explored dFC. Given the intermittent nature of epileptic discharges in SeLECTS, dFC analysis could offer a more precise understanding of how functional networks fluctuate over time and how seizure activity impacts network stability (15). Second, the interplay between different large-scale networks in SeLECTS is not yet well understood, particularly the dynamic interactions among the DMN, FPN, and DAN, which are crucial for cognitive function. Additionally, few studies have systematically examined how these brain networks evolve across different developmental stages of SeLECTS, particularly in relation to potential neuroplasticity following seizure remission. Lastly, existing rs-fMRI research has yet to incorporate functional gradient (FG) analysis, which could provide novel insights into hierarchical brain network reorganization in SeLECTS patients.

Dynamic functional connectivity (dFC) analysis captures the time-varying interactions between brain regions and is particularly suited to studying the fluctuations in brain networks in neurological diseases like epilepsy (16–18). This method can reveal temporal fluctuations in connectivity that may be overlooked in static connectivity analyses (19), which is crucial when epileptic discharges affect brain network stability (11). In SeLECTS, dFC analysis helps to understand how the brain reorganizes its networks during resting and task states, potentially serving as a compensatory mechanism to alleviate cognitive deficits (5, 20).

Functional gradient (FG) analysis provides a hierarchical perspective of brain network organization, helping to reveal how the brain transitions from lower-level motor control to higher-level cognitive integration (21). This method has been used to study hierarchical changes in the DMN and SMN, uncovering functional disruptions in various neurological and psychiatric disorders (22, 23). By combining machine learning techniques like support vector machines (SVM), FG features can help identify brain network abnormalities associated with cognitive impairments (24). However, systematic studies on the relationship between FG changes and cognitive deficits in SeLECTS are still limited.

In order to comprehensively understand the brain network abnormalities and cognitive deficits in children with SeLECTS, this study combines FG analysis and dFC analysis. FG analysis provides a global perspective of the brain network’s hierarchical structure, revealing how the brain transitions from lower-level sensory-motor control to higher-level cognitive integration. On the other hand, dFC analysis captures the temporal fluctuations of brain networks, particularly the impact of epileptic discharges on brain network stability. By combining these two methods, we can simultaneously capture the structural changes and dynamic fluctuations of brain networks, thereby providing a more comprehensive neurobiological mechanism for understanding cognitive deficits in children with SeLECTS. This combined approach has been applied in studies of other neurological and psychiatric disorders, and it helps to uncover the relationship between the disruption of functional networks and cognitive dysfunction (25, 26).

## 2 Method

### 2.1 Participants

Participants (including the SeLECTS and HC groups) were recruited from the Third Affiliated Hospital of Zunyi Medical University (First People’s Hospital of Zunyi). The diagnosis of SeLECTS was based on the criteria of the International League Against Epilepsy (27). Healthy control (HC) participants were selected from local communities, matching the SeLECTS group participants in terms of age, gender, education, and other relevant factors. Ethics number: 2024-1-713.

### 2.2 Inclusion and exclusion criteria

The study included 34 children with SeLECTS (mean age (range): 10.41 ± 1.376 years (6–16 years); 19 girls, 15 boys). The inclusion criteria were: (1) diagnosis of SeLECTS based on the criteria of the International League Against Epilepsy22; (2) no abnormalities detected on routine structural MRI; and (3) age between 6 and 16 years. The exclusion criteria were: (1) incomplete rs-fMRI scans; (2) history of other neuropsychiatric disorders; (3) history of head trauma or brain surgery; and (4) head motion exceeding 3 mm or 3° and a mean framewise displacement of 0.5 mm. Children with SeLECTS also completed the Chinese version of the Wechsler Intelligence Scale for Children on the same day as the MRI scan. Additionally, 32 healthy controls (HCs) were included (mean age (range): 11.28 ± 2.439 years (6–16 years); 14 girls, 18 boys). The inclusion criteria were: (1) no abnormalities on routine structural MRI; and (2) age between 6 and 16 years. The exclusion criteria were: (1) incomplete rs-fMRI scans; (2) history of any neurological or psychiatric disorders; (3) poor image quality; and (4) head motion exceeding 3 mm or 3° and a mean framewise displacement of 0.5 mm.

### 2.3 Image acquisition and preprocessing

MRI data were acquired using a 3.0 T MRI system (GE Healthcare, Milwaukee, WI, USA) with a 20-channel head coil. Routine MRI scans (axial T1WI, axial T2WI, and T2FLAIR) were first performed to exclude any subjects with gross structural brain abnormalities. Following this, T1-weighted three-dimensional magnetization-prepared rapid acquisition gradient echo sequences (T1WI-3D-MP RAGE) and BOLD sequences were obtained, with BOLD images acquired using echo-planar imaging. During scanning, participants were positioned supine, instructed to remain completely still, and asked to keep their eyes closed while staying awake. Foam padding, provided by the scanner manufacturer, was used to stabilize the head and reduce motion, and soft earplugs were provided to minimize noise disturbance.

The parameters for the echo-planar imaging (EPI) sequence were as follows: repetition time = 2000 ms, echo time = 30 ms, inversion time = 900 ms, flip angle = 90°, field of view = 240 mm × 240 mm, slice thickness = 4.0 mm, scan time = 420 s, and voxel size = 3.75 mm × 3.75 mm × 4 mm. High-resolution three-dimensional T1-weighted structural data were acquired with the following parameters: repetition time = 1900 ms, echo time = 2.1 ms, inversion time = 900 ms, flip angle = 15°, field of view = 240 mm × 240 mm, slice thickness = 1 mm, and scan time = 208 s.

The preprocessing of T1WI-3D-MP RAGE and BOLD images was performed using SPM (http://www.fil.ion.ucl.ac.uk/spm/), DPABI (http://www.rfmri.org/), and custom code in MATLAB (The MathWorks, Natick, MA, USA). First, the raw DICOM data were converted to NIFTI format. The first 10 functional volumes were discarded to allow for signal equilibration and environmental adaptation. The remaining 200 images were corrected for timing differences among slices within each volume through sinc interpolation and then realigned to the first volume for head motion correction. Subjects with head movement exceeding 2 mm in the x, y, or z directions and/or rotation angles greater than 2° were excluded. BOLD functional images were registered with high-resolution T1-weighted images and standardized to Montreal Neurological Institute (MNI) space (resampled voxel size = 3 mm × 3 mm × 3 mm). Noise covariates, such as cerebrospinal fluid signals, were removed, and a 6 mm full-width at half-maximum (FWHM) Gaussian smoothing kernel was applied in this study. The choice of kernel size impacts subsequent analysis: Larger kernels (e.g., 8 mm or 10 mm FWHM) improve statistical sensitivity but reduce spatial specificity. Smaller kernels (e.g., 4 mm FWHM) retain finer anatomical details but may limit the detection of network-level effects. To balance spatial resolution and noise reduction, a 6 mm FWHM kernel was selected, a commonly used standard in resting-state fMRI studies (28). Additionally, data detrending was conducted to eliminate linear or nonlinear trends from time-series data. Finally, temporal bandpass filtering was applied within the frequency range of 0.01–0.08 Hz.

### 2.4 Dynamic functional connectivity analysis

Using the sliding window method, dynamic functional connectivity (dFC) maps for each participant were obtained through DynamicBC (http://www.restfmri.net/forum/DynamicBC). In the sliding window-based dFC analysis, the window length is a key parameter. In this study, we set the window length to 50 and the sliding step size to 1, in order to investigate its impact on dynamic functional connectivity, generating a total of 90 windows (29, 30). In each sliding window, the correlation coefficients between the time series of the entire cortical brain regions and all other voxels were calculated, resulting in a series of sliding window correlation maps for each participant. To improve the normality of the correlation distribution, Fisher’s Z transformation was applied to the correlation maps. To assess the variability of dFC, the standard deviation of the Z values for each voxel was calculated, which gave the variance of the time series and correlation coefficients. K-means clustering algorithm was then used to perform clustering analysis on the functional connectivity matrices from each sliding window. The K-means algorithm involves initializing cluster centers, assigning data points to the nearest center, updating the centers, and iterating this process until the cluster centers stabilize (31). The number of clusters (k) was optimized using the elbow method to determine the optimal number of clusters. To determine the optimal number of clusters, we examined the sum of squared errors (SSE) for k values ranging from 2 to 8 and plotted the SSE curve. The analysis showed that when k = 4, the SSE exhibited an “elbow point,” indicating a balance between model complexity and variance explanation. Additionally, we calculated the Silhouette coefficient and the Calinski-Harabasz index to validate the clustering stability. Both metrics suggested that k = 4 provided the most robust separation between states while maintaining intra-cluster cohesion. Therefore, we selected k = 4. Finally, the K-means clustering analysis revealed the dynamic network patterns of the brain under different functional states and was used to compare the functional connectivity differences between the SeLECTS group and the healthy control group (HC).

### 2.5 Mapping functional gradients of the brain network

First, to construct brain network functional gradients, we extracted BOLD time series data for the seven brain networks defined by the Yeo template (32) (visual, somatomotor, dorsal attention, ventral attention, limbic, frontoparietal, and default mode networks) for both the case and HCs. Additionally, the cortex was divided into 400 regions, and the mean BOLD time series was extracted for each region. For each voxel within each brain network, Pearson correlation coefficients were calculated between the BOLD time series of each voxel and the mean time series of each cortical region, resulting in a functional connectivity matrix for each subject within each network. These functional connectivity matrices were normalized using Fisher’s r-to-z transformation. Following previous research, we used the top 10% of values in each row of the functional connectivity matrix to calculate a cosine similarity matrix, representing the connectivity similarity between each pair of voxels within each network for each subject. This process generated a similarity matrix for each participant to capture spatial microstructural patterns. Using the BrainSpace toolbox (http://github.com/MICA-MNI/BrainSpace), functional gradients were computed for the seven brain networks. The diffusion map embedding algorithm was applied to project high-dimensional data (similarity matrices) into a low-dimensional space, identifying connectivity variations between voxels (small brain regions) and extracting gradient components in descending order to explain the eigenvalues in the similarity matrix (33). Based on previous studies, we set parameters t = 0 and α = 0.5 for the gradient algorithm (α = 1 has no effect; α = 0 has maximum effect) (34, 35). A group-level gradient template was generated based on the average functional connectivity matrix across all participants. Individual gradients were then estimated and aligned to the group template using Procrustes alignment. These aligned gradients were subsequently smoothed and standardized to Z-scores for further analysis. We focused on the primary gradient component, which explains the most variance and has the highest interpretability. To capture the spatial distribution of the SMN and DMN in functional gradient space, we calculated gradient dispersion for each participant by measuring the Euclidean distances between the centroids of each network region within the aligned gradient space. This gradient space, determined by the primary functional gradients of each network, allowed us to quantify whether a brain network region is functionally integrated with or separated from other regions based on Euclidean distance calculations (35, 36).

### 2.6 Predicting the importance of FG and dFC features with machine learning models

This study employs multiple supervised learning classifiers to assess the effectiveness of FG in distinguishing SeLECTS patients from HCs. In addition to Linear Support Vector Machine (SVM) (37), we incorporated Radial Basis Function SVM (RBF SVM), K-Nearest Neighbors (KNN), Random Forest (RF), and Extreme Gradient Boosting (XGBoost) to ensure a comprehensive performance comparison. The classification models were trained using three types of features: (1) FG extracted from the DMN and SMN, and (2) dFC, derived from the sliding window method to capture temporal fluctuations in functional connectivity. Before being fed into the machine learning models, all features were standardized to ensure consistency. To enhance model robustness, Leave-One-Out Cross-Validation (LOOCV) and 5-fold cross-validation (5-fold CV) were employed during training (38). Model performance was evaluated based on accuracy, precision, sensitivity, specificity, area under the curve (AUC), and receiver operating characteristic (ROC) curves, with classification metrics calculated using a confusion matrix. The corresponding formulas are provided in Equations 1–4.


(1) $$\text{AccuracyRate}=\frac{TP+TN}{TP+FP+TN+FN}$$

(2) $$\text{Precision}=\frac{TP}{TP+FP}$$

(3) $$\text{Sensitivity}=\frac{TP}{TP+FN}$$

(4) $$\text{Specificity}=\frac{TN}{TN+FP}$$

### 2.7 Statistical analysis

A two-sample t-test was conducted to compare the functional gradient and gradient dispersion metrics between the SeLECTS and HCs, including age and gender as covariates. The statistical significance threshold was set at 0.05, and a false discovery rate correction for multiple comparisons was applied (P < 0.05, GRF correction). Pearson correlation coefficients were calculated between abnormal gradient values in the SeLECTS group and variables including age, gender, disease duration, and Wechsler Intelligence Scale scores. The significance level threshold was set at P < 0.05.

## 3 Results

### 3.1 Demographic and clinical data

A total of 38 cases were collected for the SeLECTS group, with 34 ultimately included (15 boys, 19 girls, aged 8–13 years). For the HCs, 40 children were recruited, with 32 ultimately included after excluding participants due to head motion (19 boys, 14 girls, aged 7–14 years). There were no statistically significant differences in age or gender between the SeLECTS and HCs (P > 0.05) (**Table 1**).

**Table 1:** Demographic characteristics of the sample.

	SeLECTS	HC	*t*/χ^2^	*P* Value
Number of subjects Gender (male/female)	34 15/19	32 14/19	- 1.536^a^	- 0.215
Age (years) Verbal Comprehension Perceptual Processing Working Memory Processing Speed Full Scale Years of Education Duration (years)	10.41 ± 1.376 31.41 ± 11.293 26 [23, 38] 19.56 ± 4.825 19 [14, 20] 97.68 ± 23.708 3.53 ± 1.927 1 [0.3, 1.5]	11.28 ± 2.439 - - - - - - NA	-1.816^b^ - - - - - - NA	0.074- - - - - - - NA

Data with a normal distribution are presented as mean (± standard deviation), while data with a skewed distribution are presented as median (interquartile range). Statistical significance for comparisons between groups was determined at the α=0.05 level (two-tailed). SeLECTS: Self-limited epilepsy with centrotemporal spikes; HC, Healthy controls; a denotes χ² value, b denotes t value.

### 3.2 Dynamic functional connectivity analysis

**Figure 1:**
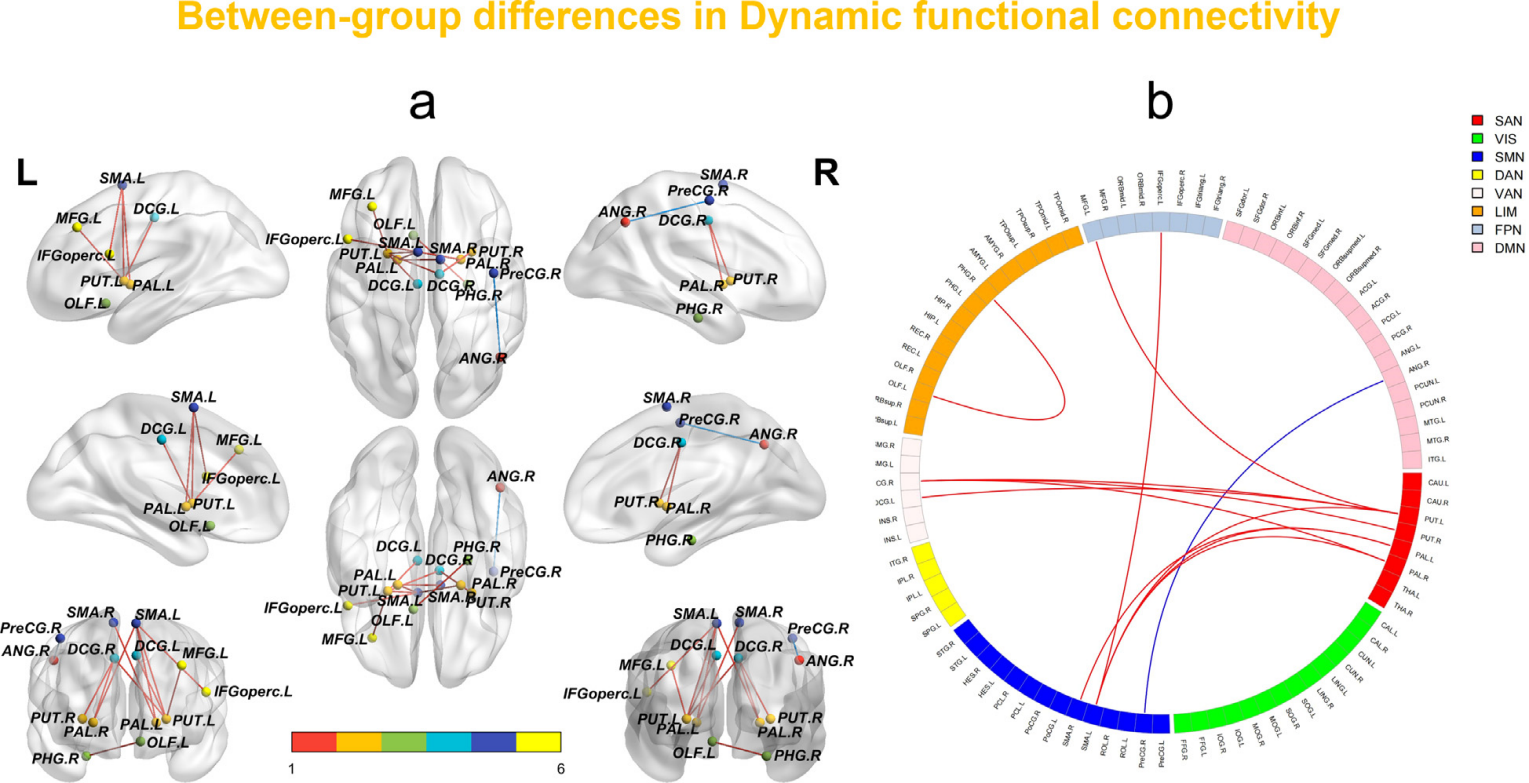
**(a, b)** In the sliding window method, network-level statistical comparisons between HCs and SeLECTS are made. Brain regions with enhanced/reduced intra- and inter-network connectivity in SeLECTS are shown in red/sky blue or dark blue. The statistical significance level was set as FDR-corrected (P < 0.05). VIS visual network, SMN sensorimotor network, DAN dorsal attention network, VAN ventral attention network, SN subcortical regions, LIM limbic network, FPN fronto-parietal network, DMN default mode network.

The dFC analysis using the sliding window method revealed increased connectivity between multiple brain regions in the SeLECTS group. Notable increases were observed in connectivity between the left inferior frontal gyrus (opercular part) and the left supplementary motor area; the left olfactory cortex and the right parahippocampal gyrus; the left supplementary motor area and the left putamen; the medial and paracingulate gyri bilaterally and the left putamen; the right medial and paracingulate gyri and the right putamen; the left supplementary motor area and bilateral globus pallidus; the right supplementary motor area and the left globus pallidus; the right medial and paracingulate gyri and the left globus pallidus; and between the left middle frontal gyrus and the putamen. In contrast, dFC between the right precentral gyrus and the left angular gyrus was decreased (**Figure 1**, **Table 2**).

**Table 2:** Between-group differences in whole-brain dynamic functional connectivity.

Brain regions	T	*p-FDR*
IFGoperc.L - SMA.L	4.254	<0.001
OLF.L - PHG.R	3.5	<0.001
PreCG.R - ANG.R	-3.472	<0.001
MFG.L - PUT.L	3.694	<0.001
SMA.L - PUT.L	4.123	<0.001
DCG.L - PUT.L	3.723	<0.001
DCG.R - PUT.L	4.761	<0.001
DCG.R - PUT.R	4.186	<0.001
SMA.L - PAL.L	4.529	<0.001
SMA.R - PAL.L	3.801	<0.001
SMA.L - PAL.R	3.779	<0.001
DCG.R - PAL.R	3.474	<0.001

Comparison of dynamic functional connectivity differences based on the sliding window method. T-value: positive value indicates enhanced connectivity, negative value indicates weakened connectivity. *p* - FDR represents the cluster size p-value corrected for false discovery rate (FDR).

**Figure 2:**
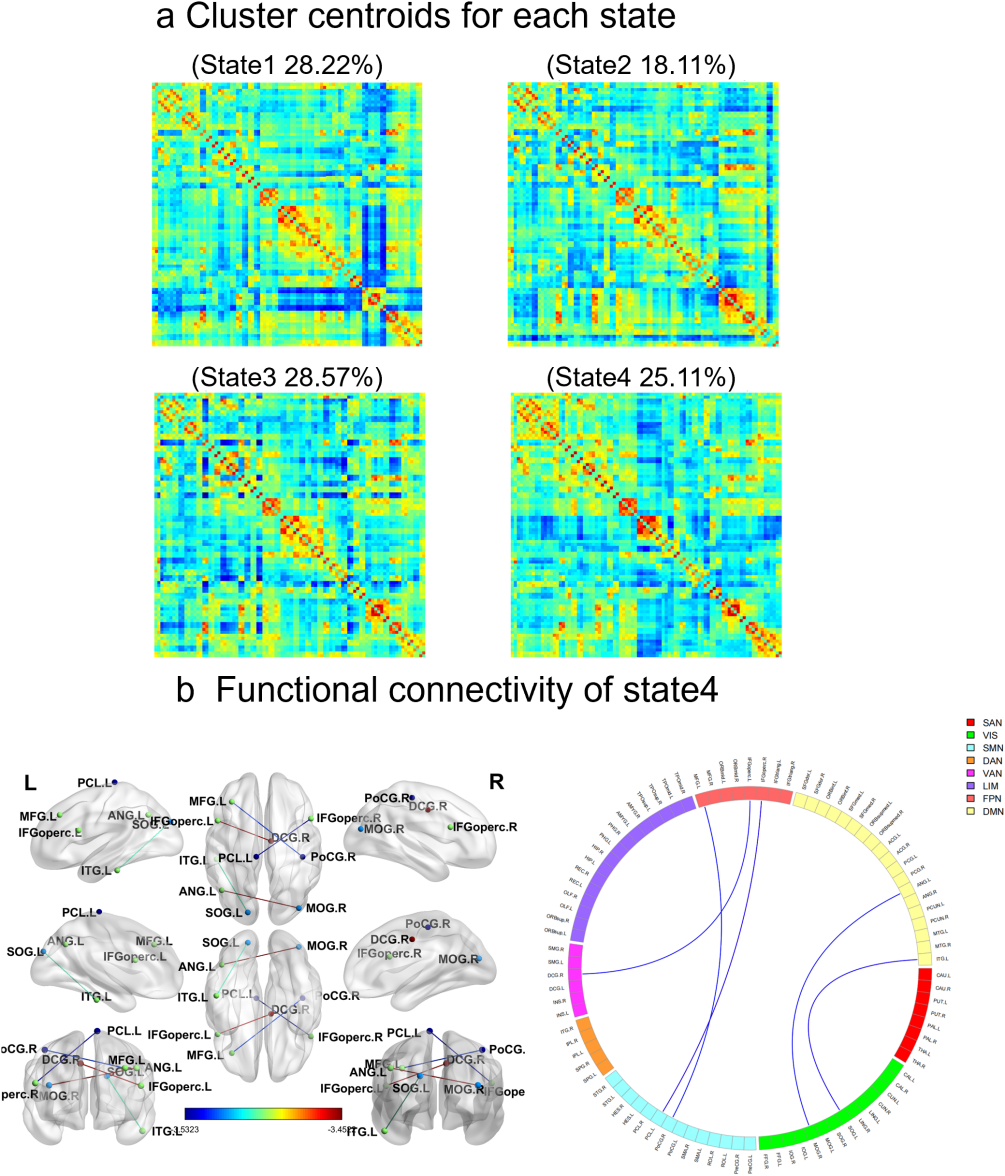
Results of the clustering analysis per state. **(a)** Cluster centroids for each state. The percentage of total occurrences is listed above each cluster median. **(b)** In state 4, there are significant differences in dynamic functional connectivity between the SeLECTS group and the HC group. The circular diagram on the left shows each square colored to represent one of the seven networks. Blue lines represent negative functional connectivity. The statistical significance level was set as FDR-corrected (P < 0.05). VIS visual network, SMN sensorimotor network, DAN dorsal attention network, VAN ventral attention network, SN subcortical regions, LIM limbic network, FPN fronto-parietal network, DMN default mode network.

In addition, the cluster analysis results revealed four states based on the optimal clustering coefficient (**Figure 2a**). In state four, the dynamic functional connectivity was reduced between the left inferior frontal gyrus (opercular part) and the right paracingulate gyrus, the left middle frontal gyrus and the right posterior central gyrus, the right middle occipital gyrus and the left angular gyrus, the right inferior frontal gyrus (opercular part) and the left paracentral lobule, as well as between the left superior occipital gyrus and the right inferior temporal gyrus (**Figure 2b**, **Table 3**). However, there were no significant differences between the two groups in terms of dynamic indicators such as the number of transitions, transition frequency, and mean dwell time.

**Table 3:** Dynamic functional connectivity differences in State 4.

Brain regions	T	*p-FDR*
IFGoperc.L - DCG.R	-3.456	<0.001
MFG.L - PoCG.R	-3.52	<0.001
MOG.R - ANG.L	-3.452	<0.001
IFGoperc.R - PCL.L	-3.532	<0.001
SOG.L - ITG.L	-3.498	<0.001

Based on K-means clustering analysis, the comparison of dynamic functional connectivity differences in State 4. T-value: Positive values indicate enhanced connectivity, negative values indicate weakened connectivity. *p* - FDR represents the cluster size p-value corrected for false discovery rate (FDR).

### 3.3 Functional gradient of the somatomotor network and default mode network

**Figure 3:**
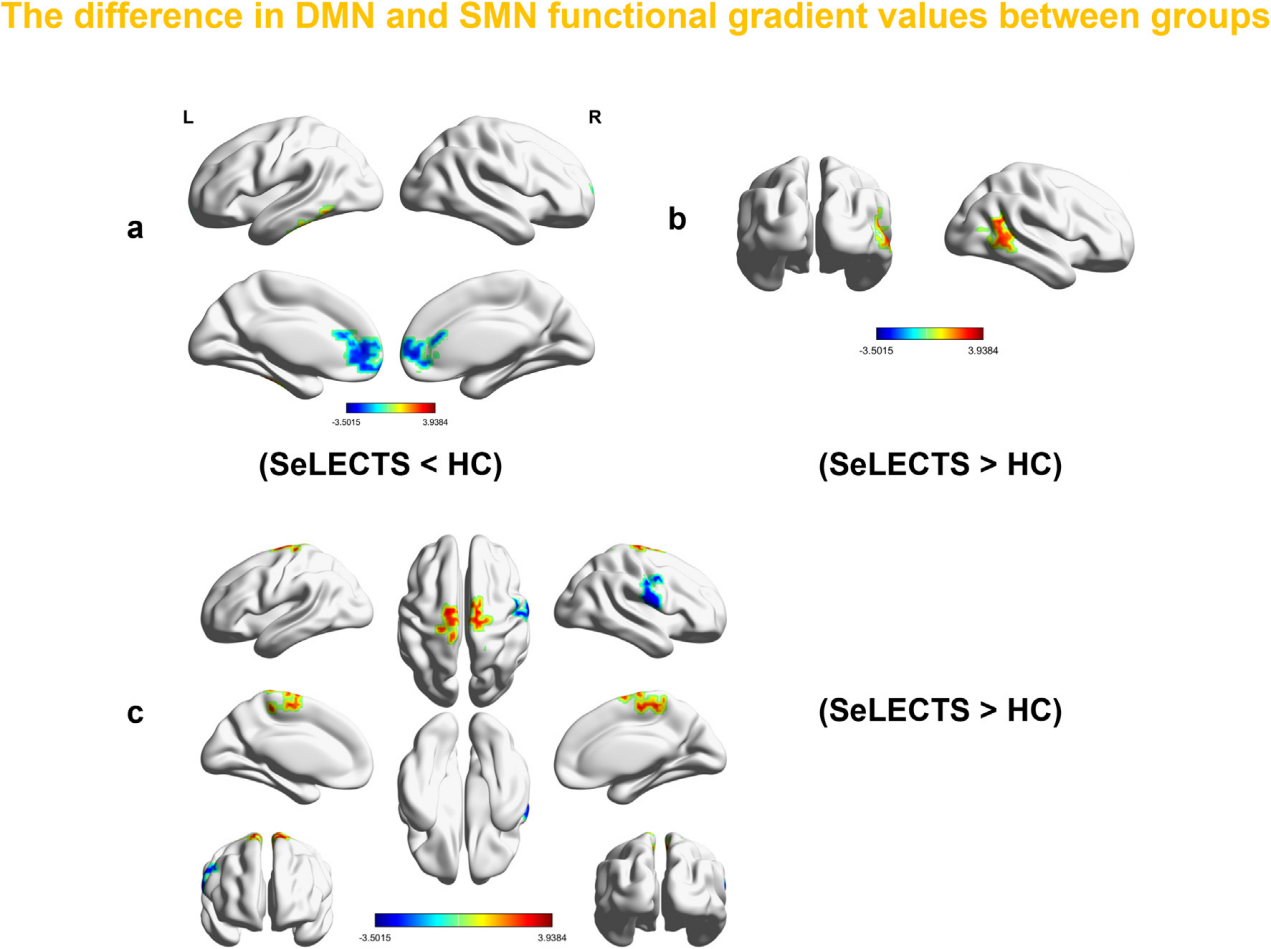
The regions with differences in the first and second gradients of the DMN between all SeLECTS patients and HCs in this study. Surface rendering was generated using BrainNet Viewer. **(a)** In the first gradient of the DMN, SeLECTS patients showed a decreased gradient score in the bilateral SFGdor and an increased score in the ITG.L. **(b)** In the second gradient of the DMN, an increased gradient score was observed in the MTG.R of the SeLECTS group **(c)** The regions showing differences in the primary gradient of the SMN between all SeLECTS patients and HCs in this study, an increased score was observed in the bilateral SMA, while decreased scores were found in the right PreCG (all between-group differences were assessed using two-sample t-tests, GRF correction (voxel *p* value < 0.001 and cluster *P* value<0.05)). SeLECTS Central Temporal Spiking Self-Limited Epilepsy, DMN default mode network, SFGdor dorsolateral superior frontal gyrus, ITG.L left inferior temporal gyrus, MTG.R right middle temporal gyrus, SMN Sensorimotor Network, SMA. supplementary motor area, PreCG precentral gyrus.

Voxel-level analysis of functional gradients in the DMN and SMN revealed significant differences between SeLECTS patients and HCs. Specifically, in the first gradient of the DMN, SeLECTS patients showed a decreased gradient score in the bilateral dorsolateral superior frontal gyrus and an increase in the left inferior temporal gyrus (**Figure 3a**, **Table 4**). In the second gradient of the DMN, an increased score was observed in the right middle temporal gyrus in the SeLECTS group (**Figure 3b**, **Table 4**). In the primary gradient of the SMN, an increased score was observed in the bilateral supplementary motor area, while decreased scores were found in the right precentral gyrus (**Figure 3c**, **Table 4**).

**Table 4:** Brain regions with significant differences in gradient scores between SeLECTS and HCs.

Brain regions (AAL3)	Voxel s, n	MNI coordinates, mm (x, y, z)			Peak *T* values
Gradient 1					
right Frontal Sup	512	18	54	9	-3.2075
left Frontal Sup	533	-16	57	9	-3.2395
left Temporal Inf right Supp Motor Area left Supp Motor Area right Precentral	201 515 525 383	-51 15 -10 27	-33 -6 -8 -18	-21 69 71 57	3.3219 3.9384 3.9215 3.3136
Gradient 2					
right Temporal Mid	337	60	-54	12	4.5453

GRF correction, two-tailed test (voxel *p* value < 0.001 and cluster *P* value<0.05), MNI, Montreal neurological institute; AAL3, automated anatomical labeling; GRF, Gaussian random field.

### 3.4 Predicting the importance of functional gradient features with machine learning models

**Figure 4:**
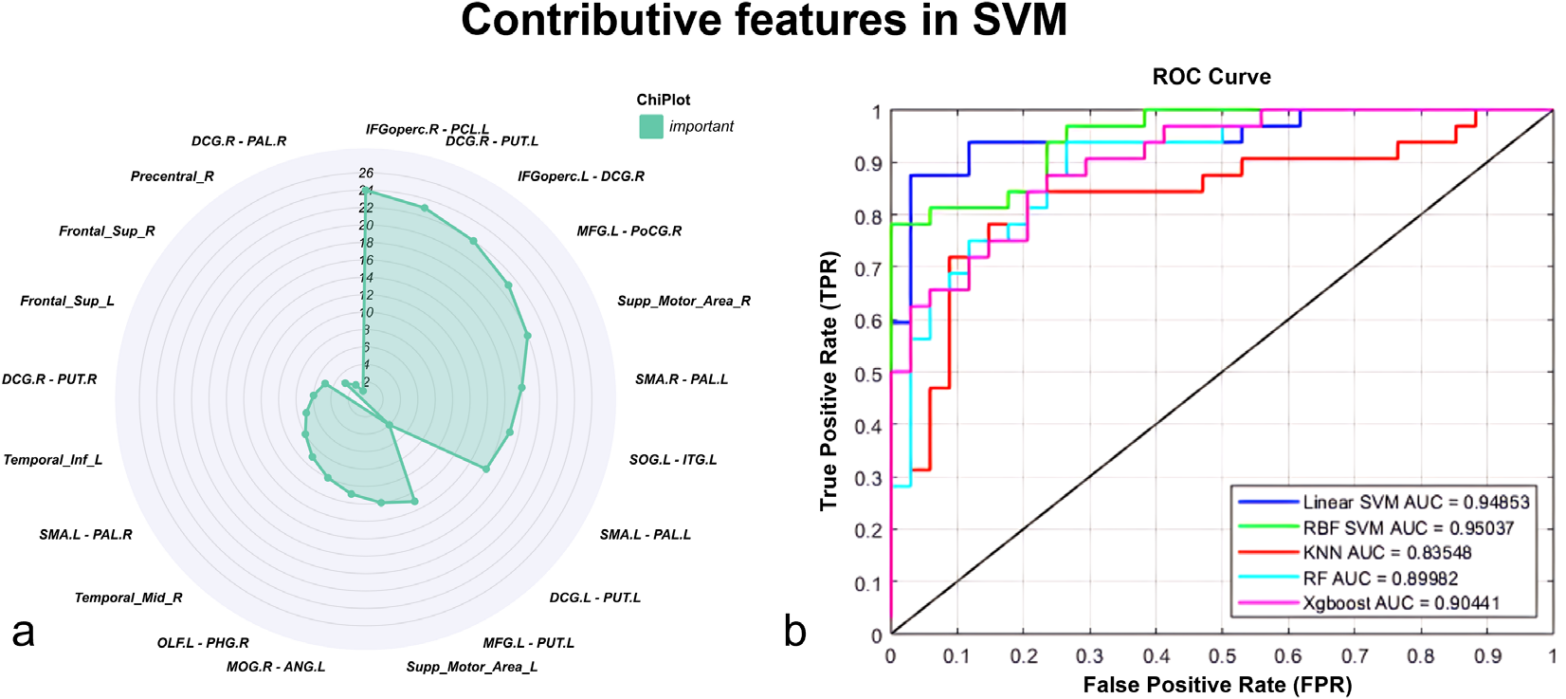
**(a)** The radar chart illustrates the importance of 24 FG and dFC features in distinguishing the SeLECTS group from healthy controls based on SVM analysis. **(b)** Feature curves of the 24 features across multiple machine learning models, including Linear SVM, RBF SVM, KNN, RF, and XGBoost. Linear Support Vector Machine Linear SVM, Radial Basis Function SVM RBF SVM, K-Nearest Neighbors KNN, Random Forest RF, Extreme Gradient Boosting XGBoost.

To comprehensively evaluate the performance of FG and dFC in SeLECTS classification, we employed multiple machines learning models, including Linear SVM, RBF SVM, KNN, RF, and XGBoost. All models achieved AUC values above 0.80, ind.icating stable and reliable classification performance. Among them, Linear SVM demonstrated the best performance, achieving an Area Under the Curve (AUC) of 0.949, sensitivity of 0.844, specificity of 0.941, and accuracy of 0.894, suggesting strong discriminative power in SeLECTS prediction (**Figure 4b**).

The model results further indicated that the dFC between the right opercular part of the IFGoperc.R and PCL.L played a crucial role in distinguishing SeLECTS patients from healthy controls (HCs), highlighting significant differences in brain regions between the two groups (**Figure 4a**).

### 3.5 Clinical association of functional gradients

**Figure 5:**
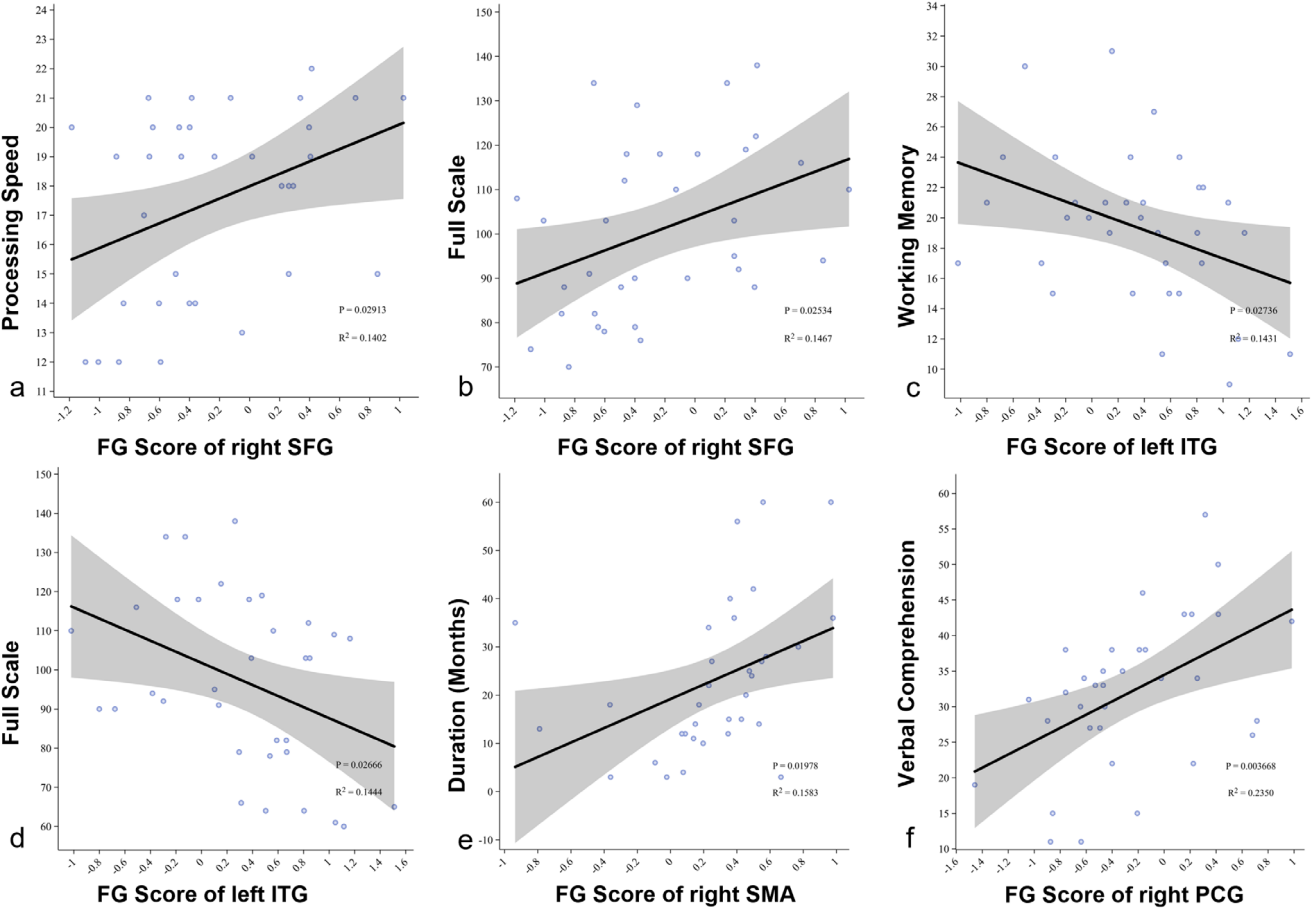
**(a, b)** Pearson correlation analysis showing the relationship between the gradient score of the right superior frontal gyrus and processing speed and full-scale IQ in SeLECTS patients; **(c, d)** Pearson correlation analysis showing the relationship between the gradient score of the left inferior temporal gyrus and working memory and full-scale IQ in SeLECTS patients; **(e)** Pearson correlation analysis showing the relationship between the gradient score of the right supplementary motor area and duration in SeLECTS patients; **(f)** Pearson correlation analysis showing the relationship between the gradient score of the left precentral gyrus and verbal IQ in SeLECTS patients. SFG dorsolateral superior frontal gyrus, ITG inferior temporal gyrus, SMA.R right supplementary motor area, PCG precentral gyrus.

Correlation analysis showed that in the SeLECTS group, the gradient value of the right dorsolateral superior frontal gyrus in the first default mode network (DMN) was positively correlated with processing speed (R² = 0.140, p = 0.029) and full-scale IQ (R² = 0.351, p = 0.025) (**Figures 5a, b**). In contrast, the gradient value of the left inferior temporal gyrus was negatively correlated with working memory (R² = 0.143, p = 0.027) and full-scale IQ (R² = 0.144, p = 0.027) (**Figures 5c, d**). Within the sensorimotor network (SMN), the gradient value of the right supplementary motor area was positively correlated with disease duration (R² = 0.158, p = 0.020) (**Figure 5e**), while the left precentral gyrus was positively correlated with verbal IQ (R² = 0.235, p = 0.003) (**Figure 5f**).

## 4 Discussion

In this study, the sliding window method was used for dFC analysis, revealing significant changes in the FC of multiple brain regions between children with SeLECTS and HC. In the SeLECTS group, dFC between the left precentral gyrus and the bilateral supplementary motor area (SMA), as well as the globus pallidus, was significantly enhanced. These regions are primarily involved in motor control and executive functions, with the precentral gyrus and SMA playing key roles in motor regulation (39, 40). The increase in dFC suggested that children with SeLECTS may rely on stronger synchronization within the motor network, which could be a compensatory mechanism for epileptic discharges to maintain or optimize motor function. However, this enhancement in functional connectivity may lead to excessive synchronization in brain activity, potentially impairing the flexible coordination between brain regions, which can negatively affect cognitive and behavioral functions (41). The enhanced connectivity between the precentral gyrus and SMA is crucial for motor control and the execution of complex motor tasks, and this enhancement helped improve motor control and task performance (42). Liebrand et al. (43)pointed out that increased SMA activity is closely related to the efficiency of motor planning and execution, suggesting that the enhanced connectivity between SMA and the precentral gyrus may assist in learning and controlling complex motor behaviors.

In terms of cognitive control networks, the SeLECTS group showed enhanced dynamic functional connectivity between the left middle frontal gyrus and the putamen, as well as between the right medial gyrus, the paracingulate gyrus, and the left putamen, indicating a significant increased in cross-network interactions. The middle frontal gyrus plays a key role in executive functions and attention control, while the putamen is essential for action inhibition and behavioral regulation (44, 45). Li et al. (46) found that individuals with long-term motor training showed enhanced connectivity in the cortico-basal ganglia circuit, and this cross-network integration helps optimize complex motor control functions. Similarly, in patients with cognitive impairments, enhanced connectivity between the middle frontal gyrus and regions such as the putamen helps manage additional cognitive load, particularly in attention and executive functions (47). However, the excessive enhancement of cross-network connectivity might have led to over-synchronization between different brain networks, affecting the integration and coordination of information across regions, thereby impaired the efficient execution of cognitive functions.

In the SeLECTS group, the dFC between the right precentral gyrus and the left angular gyrus was significantly reduced, suggesting decreased coordination between the SMN and the DMN. This result aligned with findings from the functional gradient analysis, which indicates that epilepsy disrupts the integration between motor control and cognitive functions (such as language and memory), leading to a “blurring” or “mixing” of these functions (48). In children with epilepsy, this mixed effect may reduce the efficiency of cognitive resource allocation between motor and higher functions, negatively impacting their behavioral and cognitive performance. Additionally, the weakening of dynamic functional connectivity might have led to reduced synchronization between brain networks, further hindering the efficiency and flexibility of information processing. These changes in dynamic functional connectivity in SeLECTS children not only reflect alterations in synchronization between different brain regions but also point to potential mechanisms of over-synchronization or decreased synchronization in brain function, which could have a profound effect on both cognitive and motor functions.

Based on the results of the clustering analysis, the SeLECTS group exhibited reduced dynamic functional connectivity between the left inferior frontal gyrus (insula) and the right posterior cingulate cortex, the left middle frontal gyrus and the right posterior central gyrus, the right occipital middle gyrus and the left angular gyrus, the right inferior frontal gyrus (insula) and the left posterior central gyrus, and the left superior occipital gyrus and the right inferior temporal gyrus in state four, this is similar to the findings of Li et al. (30) study. These findings further support the conclusion of diminished coordination between specific brain networks in SeLECTS children, particularly between regions involved in motor control, cognitive processing, and sensory integration. For example, the reduced connectivity between the left inferior frontal gyrus (insula) and the right posterior cingulate cortex may reflect a loss of coordination between the motor and default mode networks, which could further exacerbate the separation between motor control and cognitive functions (49). Additionally, the reduced connectivity between the left middle frontal gyrus and the right posterior central gyrus may indicate a decrease in cross-network interactions between the frontal and sensory-motor networks, potentially impairing the ability of SeLECTS children to integrate information in complex tasks.

However, despite the changes in dynamic functional connectivity between certain brain regions, there were no significant differences between the two groups in terms of dynamic indicators such as transition count, transition frequency, and average dwell time. This suggests that, although the functional connectivity patterns in SeLECTS children underwent significant changes, the overall stability of dynamic transitions remained unaffected. This phenomenon might have reflected the brain’s adaptive regulatory mechanisms to accommodate changes in functional connectivity, such that despite significant alterations in connectivity between certain brain regions, the stability of the overall dynamic network is maintained (50), thereby somewhat mitigating the negative impact of functional abnormalities on behavior and cognitive function.

Functional gradients represent the continuous transition of brain functions from primary sensorimotor areas to higher-order cognitive regions. This study reveals distinct functional gradient alterations within the default mode network (DMN) and sensorimotor network (SMN) in children with self-limited epilepsy with centrotemporal spikes (SeLECTS), providing deeper insights into functional connectivity patterns in SeLECTS. Previous rs-fMRI studies have shown significant abnormalities in both sensorimotor and DMN regions among children with SeLECTS (7, 15, 51–54). Through analysis of functional gradients within the DMN and SMN, this study observed that the functional distribution patterns in SeLECTS children differ significantly from those in HCs. These findings suggested that the observed functional gradient changes in the DMN and SMN may contribute to understanding the impact of SeLECTS on cognitive and motor functions, offering new insights into the neural mechanisms underlying this condition.

In the first gradient of the DMN, children with SeLECTS showed a decreased gradient score in the bilateral dorsolateral superior frontal gyrus and an increased gradient score in the left inferior temporal gyrus. The superior frontal gyrus is located in the frontal lobe and is involved in higher cognitive functions such as working memory and self-awareness (55). In contrast, the inferior temporal gyrus, located in the lower part of the temporal lobe, primarily supports semantic memory, visual processing, and semantic understanding, and is linked with language processing and emotional regulation (56). Studies indicate that gradient changes within the DMN in children with SeLECTS may affect the normal integration of semantic memory, language, and attention. In healthy individuals, the DMN gradient generally shows a separation from sensorimotor regions to heteromodal regions (such as those involved in language and semantic processing) (22). However, in children with SeLECTS, these gradient abnormalities may lead to a reduced gradient score in the right superior frontal gyrus, suggesting that the function of this region is gradually diverging from the DMN’s core role, which in turn may impact its cooperative role in higher cognitive functions. Furthermore, the DMN’s separation effect in semantic integration is more pronounced in heteromodal apex areas, particularly in tasks involving the integration of visual and emotional cues, highlighting the importance of the DMN in information integration and higher cognitive functions (57).

Notably, in the second gradient of the DMN, children with SeLECTS showed an increased gradient score in the right middle temporal gyrus, this is similar to the findings of Jiang et al. (58), where abnormalities in dReHo were observed in the middle temporal gyrus. This region is primarily involved in language, memory, and emotional processing, and the increase in its gradient score may reflect local functional reorganization within the DMN to adapt to the effects of SeLECTS on language and emotional integration. Such compensatory changes may reflect the organizational principles of the DMN in integrating long-term semantic knowledge and novel concepts within higher cognitive functions (59).

In the first gradient of the sensorimotor network (SMN), the gradient score of the right SMA was increased, while scores in the bilateral precentral gyrus were decreased. Research by Ruan et al. highlights that the SMA’s connectivity strength within the SMN plays a critical role in motor control, with an increased gradient score in this region indicating its importance in motor function (60). Additionally, Ji et al. demonstrated that inhibitory interventions targeting the SMA significantly impact its connectivity patterns within the SMN, underscoring the central role of the SMA in motor control (61). Thus, the increase in the SMA gradient score may suggest a potential association between epileptic discharges on motor control regions.

In contrast, the decreased gradient scores in the precentral may reflect reduced integration within the SMN and potentially increased connectivity with higher-order cognitive networks like the DMN. This change aligns with findings by Hartwigsen et al. (62), who observed that neural networks exhibit adaptive plasticity through dynamic reorganization after injury. This reallocation of resources through cross-network interactions helps maintain functional stability, similar to compensatory mechanisms observed in the brain after injury, where cross-network cooperation enables the compensation of higher functions. This suggests a gradual blurring of boundaries between motor and cognitive networks in children with SeLECTS.

The correlation analysis further supports these findings, showing that the gradient score of the right dorsolateral prefrontal cortex is positively correlated with processing speed and full-scale IQ, emphasizing the importance of this region in information processing. In contrast, the gradient score of the left inferior temporal gyrus is negatively correlated with working memory and IQ, suggesting that abnormalities in this region may increase cognitive load. The functional gradient changes in the right middle temporal gyrus and left inferior temporal gyrus may reflect the impact of SeLECTS on language and memory functions and suggest potential adaptive mechanisms of the DMN in cognitive and emotional processing. Analysis of the sensorimotor network (SMN) shows that the gradient score of the right supplementary motor area (SMA) is positively correlated with disease duration, while the left postcentral gyrus is positively correlated with language IQ, indicating the long-term effects of epilepsy on motor control regions and revealing the potential role of the SMN in maintaining language functions.

This study used SVM analysis to identify specific abnormal brain network connectivity in children with SeLECTS. The results showed that the dFC between the right opercular part of the IFGoperc.R and the PCL.L played a crucial role in distinguishing SeLECTS patients from HCs. This connection achieved an AUC of 0.949, sensitivity of 0.844, specificity of 0.941, and accuracy of 0.894, demonstrating strong discriminative power. Previous research has shown that the right inferior frontal gyrus is involved in language processing, cognitive control, and executive function, while the paracentral lobule is closely related to somatosensory processing (63, 64). Epileptic discharges in SeLECTS patients may disrupt coordination between these regions, leading to impaired integration of motor control and cognitive functions (65). Recursive Feature Elimination (RFE) analysis further confirmed that dFC in this connection serves as a key feature, indicating its role in brain network reorganization in SeLECTS and its potential impact on higher-order cognitive function and adaptability in complex tasks.

To ensure the robustness of our findings, we applied multiple machines learning models, including Linear SVM, RBF SVM, KNN, RF, and XGBoost, to comprehensively evaluate the role of FG and dFC in SeLECTS classification. The results demonstrated that all models achieved AUC values above 0.80, indicating stable classification performance and reliable results. Notably, even across different machine learning algorithms, the dFC between IFGoperc.R and PCL.L consistently remained a key distinguishing feature, reinforcing its reliability as a robust neuroimaging biomarker. These findings suggest that this connection holds potential clinical value for accurately differentiating SeLECTS patients from healthy individuals.

## 5 Limitations

First, the small sample size may limit the generalizability of the findings. A small dataset increases the risk of overfitting, which could lead to an overestimation of model performance. Future studies should include larger and more diverse samples to improve the robustness and applicability of the results. Second, this study used single time-point imaging analysis, which restricted insights into the dynamic progression of epilepsy. As SeLECTS is a self-limited epilepsy syndrome, longitudinal changes in brain networks could provide valuable information on how neural connectivity evolves over time. Future research should incorporate longitudinal and multimodal imaging approaches to validate these findings and explore the neurodevelopmental characteristics of SeLECTS. Third, the SVM classification model lacked validation on an independent dataset, making it uncertain whether the model can reliably classify different patient populations. Future studies should incorporate external dataset validation to further confirm its diagnostic value. Finally, this study did not fully account for the potential impact of anti-epileptic medications on brain functional connectivity. Anti-epileptic drugs (AEDs) might have altered functional connectivity and network dynamics, potentially influencing the interpretation of results. Future research should control for or systematically investigate the effects of different pharmacological treatments to distinguish epilepsy-related changes from medication-induced alterations. Addressing these limitations in future studies will enhance the understanding of SeLECTS-related brain network alterations and improve the clinical utility of functional gradient analysis.

## 6 Conclusion

This study utilized functional FG, dFC, and SVM methods to identify significant abnormalities in the DMN and SMN of SeLECTS patients, particularly in the dFC between the IFGoperc.R and the PCL.L. These findings suggest the potential of dFC as a neuroimaging biomarker for cognitive assessment in epilepsy, offering new insights into the neurobiological mechanisms of SeLECTS.

## Data Availability

The raw data supporting the conclusions of this article will be made available by the authors, without undue reservation.
